# Physicochemical and Stability Study of Chitosan-Coated Nanoliposomes: Effects of Polymer Molecular Weight and Ultrahigh Pressure Homogenization-UHPH

**DOI:** 10.3390/polym18151853

**Published:** 2026-07-29

**Authors:** Mariana Sierra, Sandra Navarro-Gallón, Ana L. Giraldo, Yhors Ciro, Constain H. Salamanca

**Affiliations:** 1 Grupo Natura, Departamento de Ciencias Farmacéuticas y Químicas, Facultad Barberi de Ingeniería, Diseño y Ciencias Aplicadas, Universidad Icesi, Calle 18 No. 122-135, Cali 760031, Colombia; marisierraa29@gmail.com; 2 Grupo de Investigación Cecoltec, Cecoltec Services, Cra. 43A #18 Sur-135, Medellín 050022, Colombia; snavarro@cecoltecservices.com; 3 Departamento de Farmacia, Facultad de Ciencias Farmacéuticas y Alimentarias, Universidad de Antioquia, Calle 70 No. 52-21, Medellín 050010, Colombia; liliana.giraldo@udea.edu.co; 4 Grupo de Investigación en Química y Biotecnología (QUIBIO), Facultad de Ciencias Básicas, Universidad Santiago de Cali, Cali 760035, Colombia

**Keywords:** chitosan, layer-by-layer, molecular weight, nanoliposomes, physicochemical characterization, ultra-high-pressure homogenization

## Abstract

This study evaluated the effect of chitosan with a high degree of deacetylation (75–85%) and three different molecular weights—low (50–190 kDa), medium (190–310 kDa), and high (310–375 kDa)—on the coating of vesicular systems. Chitosan solutions were characterized in acidulated aqueous medium by determining changes in pH, electrical conductivity, zeta potential, surface tension, viscosity, transmittance, particle size, and polydispersity index (PDI) with respect to polymer concentration. Subsequently, nanoliposomes were developed using an ethanol injection method assisted by ultra-high-pressure homogenization (UHPH) prior to coating. The characterization of the vesicular systems involved particle size analysis, polydispersity index (PDI), and zeta potential, which were evaluated at zero time and at the fourth week prior to storage at 4 °C and 40 °C. The physicochemical characterization of chitosan solutions displayed an inflection point at 1 × 10^−3^ M, which was taken as the coating concentration. In general, non-coated liposomes ranged between 150 and 200 nm, with low polydispersity (<0.3) and negative zeta potentials around ~−43 mV. Chitosan coating significantly increased particle size and PDI, while decreasing zeta potential values, but within the same negative value range, suggesting a slight interfacial coating effect. Finally, the systems subjected to UHPH and coated with high- and medium-molecular-weight chitosan showed interesting stabilization against storage conditions in the thermal stress tests.

## 1. Introduction

Currently, nanotechnology is an important tool for developing advanced systems to encapsulate and control the release of active compounds, with nanoliposomes being among the most relevant [1,2,3]. These vesicles, formed by the self-assembly of phospholipid bilayers [4], can carry both hydrophilic and hydrophobic molecules, protecting them from degradation and improving their functional properties [5,6]. Despite these advantages, nanoliposomes often show physicochemical instability, including aggregation, fusion, and leakage during storage and processing [7]. Solving these problems is essential for their application in sectors such as pharmaceuticals, cosmetics, and food.

One way to improve the stability of vesicular systems is to modify their surface by coating them with polymers, with chitosan being one of the most widely used. This cationic polysaccharide is well known for its biocompatibility, low toxicity, and biodegradability, as well as for its versatility in terms of degree of deacetylation and molecular weight [5,8,9,10,11]. In phospholipid-based vesicular systems, the positive charge of chitosan interacts strongly with the negative phosphate groups of the phospholipid head, forming a protective layer on the liposomal surface. This layer can be partial or complete, arranged as monolayers or multilayers [12]. Such coating improves colloidal stability by reducing vesicle aggregation, making chitosan-coated liposomes (chitosomes) a useful option for encapsulating active compounds [13,14]. In addition, chitosan provides antimicrobial and mucoadhesive properties, which expand its applications in different industrial sectors [15,16].

Regarding the molecular weight of chitosan, it has been identified as a key factor that can affect several physicochemical properties, and in the specific case of coating, it can influence the density, rigidity, and flexibility of the vesicular system [17]. In this way, low-molecular-weight chitosan provides gradual stabilization, ranging from slight to moderate, while medium and high-molecular-weight chitosan tend to form more compact coatings with greater resistance to surface desorption [18]. Thus, in line with the above, the characterization of chitosan in aqueous medium prior to the layer-by-layer coating process is a fundamental step for optimizing this procedure, since the polymer tends to deposit more effectively on the vesicle surface rather than aggregating with other polymer chains [19,20,21].

On the other hand, ultra-high pressure homogenization (UHPH) [22,23], a technique widely recognized for its ability to reduce particle size and polydispersity in emulsion systems, has also been applied to liposomal systems [13]. Nevertheless, it is important to note that only a few studies have addressed this topic, and those available suggest that this technique significantly affects liposome stability [15,16]. Therefore, it is necessary to expand research in this field, particularly regarding the influence of polymer molecular weight on the coating of vesicular systems processed with and without UHPH.

Finally, several recent studies in this field are worth mentioning, as they confirm the versatility of chitosan-coated liposomes. Gil-Gonzalo et al. (2024) showed that chitosomes improved the encapsulation and release profiles of ciprofloxacin and etoposide, enhancing stability and therapeutic efficacy [7]. Wen et al. (2024) reported that moringin-loaded chitosomes exhibited strong antibacterial activity against *Staphylococcus aureus*, disrupting cell membranes and inhibiting biofilm formation [14]. Similarly, Chen et al. (2022) demonstrated that curcumin-loaded chitosomes prepared by ethanol injection and high-pressure processing achieved superior thermal stability and antimicrobial activity [13]. Shirnoush et al. (2025) highlighted the role of chitosan molecular weight in stabilizing ferrous sulfate-loaded nanoliposomes [18], while de Barros et al. (2025) explored intranasal delivery of ghrelin using chitosomes, achieving enhanced brain bioavailability [24]. Collectively, these studies underscore the broad potential of chitosan-coated liposomes as multifunctional carriers.

As mentioned previously, this study focused on evaluating the behavior of three chitosan polymers with low, medium, and high molecular weight in aqueous medium to determine the concentration at which they exist as individual chains and polymeric aggregates. Subsequently, nanoliposomes were prepared using the ethanol injection method with and without UHPH treatment and then coated using the layer-by-layer method. Finally, their stability under storage conditions (4 °C and 40 °C) was assessed over a period of four weeks. It is expected that these findings will contribute to the rational design of chitosan-coated vesicular systems, reinforcing the potential of coated liposomes as robust platforms for the encapsulation and release of bioactive compounds.

## 2. Materials and Methods

### 2.1. Materials

Low- (50–190 kDa), medium- (190–310 kDa), and high (310–375 kDa)-molecular-weight chitosan polymers, all with a degree of deacetylation of 75–85% (Sigma-Aldrich, St. Louis, MO, USA), were employed as received, without further purification or chemical modification. Soy lecithin of low purity grade (Lp-SBL) was obtained from Laboratorios San Jorge (Cali, Colombia). According to its technical specifications, this material contains approximately 90% phospholipids, predominantly phosphatidylcholine (~50%), inositol phosphatides (~30%), and phosphatidylethanolamine (~10%), with the remaining ~10% corresponding to non-phospholipid impurities [25]. Ultrapure water was supplied from an Elix Essential Millipore^®^ purification system, Darmstadt, Germany, with a mean conductivity value of ~0.050 μS/cm. Potassium phosphate monobasic and potassium phosphate dibasic, sodium hydroxide pellets, and glacial acetic acid (1 N) were all purchased from Sigma-Aldrich (Merck KGaA, Darmstadt, Germany). Phosphate-buffered saline (PBS, pH 7.2, ionic strength 10 mM) was freshly prepared using ultrapure water and the above salts. All materials were used as received, without further purification.

### 2.2. Physicochemical Characterization of Chitosan in Aqueous Solution

Chitosan polymers of different molecular weights were characterized in aqueous medium at concentrations ranging from $1\times 10^{-6}\;M\;$ to $1\times 10^{-2\;}M$, expressed relative to the average monomeric unit. Since chitosan is composed of D-glucosamine (GlcN, MW ≈ 161 g/mol) and N-acetyl-D-glucosamine (GlcNAc, MW ≈ 203 g/mol) and exhibits a degree of deacetylation (DD) of 75–85%, the effective molecular weight of its monomeric unit was determined to be ~169 g/mol for DD ≈ 80%. The solvent consisted of 1% acetic acid solution. Physicochemical characterization included particle size, polydispersity, turbidimetry, zeta potential, conductivity, pH, surface tension, and viscosity. All measurements were performed in triplicate using freshly prepared samples to ensure reproducibility and minimize variability.

#### 2.2.1. pH, Electrical Conductivity, and Zeta Potential

pH was determined with an Ohaus ST2100-F pH meter (Ohaus, Parsippany, NJ, USA), calibrated with standard buffers at pH 4.0 and 7.0, while conductivity and Zeta potential were measured with a Zetasizer Nano ZSP (Malvern Instruments, Worcestershire, UK) using a folded capillary cell (DTS 1070). Electrophoretic mobility was converted to zeta potential using the Smoluchowski equation [26].

#### 2.2.2. Surface Tension

Surface tension was measured using an OCA15EC tensiometer (Dataphysics Instruments, Filderstadt, Germany), equipped with a high-resolution video camera and controlled by SCA22 software (version 4.5.14). A 500 µL sample was dispensed through a dosing syringe, and the drop profile was recorded. The Young–Laplace equation [27] was applied to calculate surface tension.

#### 2.2.3. Viscosity

Viscosity was determined using a microVISCTM instrument (RheoSense Inc., San Ramon, CA, USA) [28] with chip A05 (0–100 cP). Each measurement required 400 µL of sample, and flow was controlled to ensure laminar conditions. The instrument provided absolute viscosity based on pressure-driven flow through a microfluidic channel.

#### 2.2.4. Turbidimetry

Transmittance was measured using a UV–Vis spectrophotometer (Shimadzu UV-1800, Kyoto, Japan) at 600 nm, a wavelength selected to minimize absorption and maximize sensitivity to scattering phenomena.

#### 2.2.5. Particle Size and Polydispersity

Particle size and polydispersity index (PDI) were measured using a Zetasizer Nano ZSP (Malvern Instruments, Worcestershire, UK) equipped with a red He/Ne laser operating at 633 nm. Measurements were performed by dynamic light scattering (DLS) at a scattering angle of 173° and a controlled temperature of 20 °C, employing a quartz flow cell (ZEN0023). DLS evaluates the diffusion of particles under Brownian motion, generating a correlation function that follows the Stokes–Einstein relationship [29], which allows calculation of the hydrodynamic diameter and distribution parameters. The cumulative correlation function was fitted with a simple exponential model to obtain the mean particle size (z-average diameter) and the PDI, while multiple exponential fitting combined with non-negative least squares (NNLS) or constrained regularization (CONTIN) provided the particle size distribution [30]. In this study, particle size is reported as the z-average diameter, and PDI values are interpreted within the range of 0 to 1, where values closer to 0 indicate monodisperse systems and values approaching 1 reflect broad size distributions. For DLS analysis, samples were diluted at a ratio of 1:10.

### 2.3. Preparation and Characterization of Chitosan-Coated Nanoliposomes

**Figure 1:**
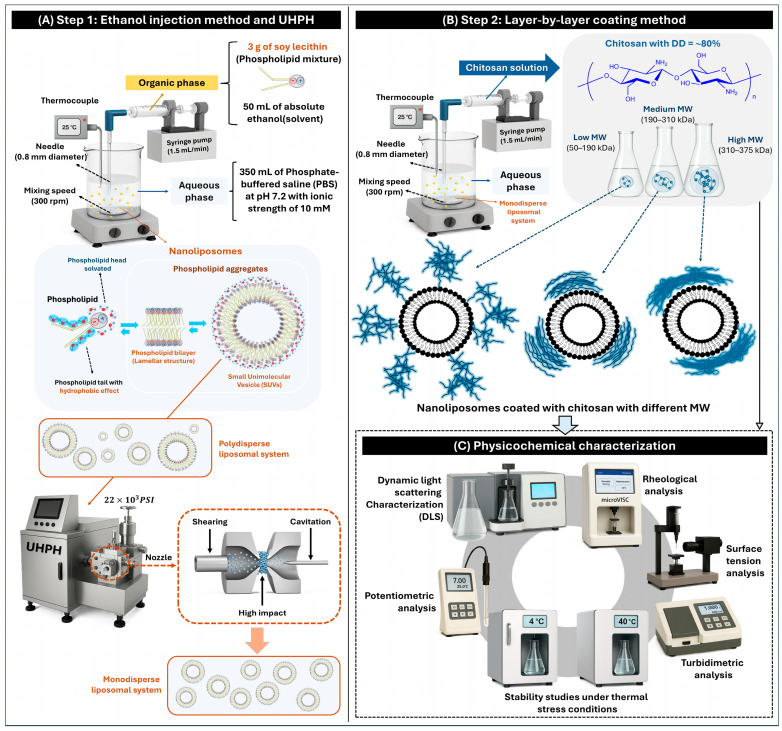
Schematic representation of the preparation and characterization of nanoliposomal systems. The diagram illustrates: (**A**) the ethanol injection method followed by UHPH to obtain monodisperse liposomes; (**B**) the subsequent layer-by-layer deposition of chitosan with different molecular weights to generate coated nanoliposomes; and (**C**) the physicochemical characterization by dynamic light scattering (DLS) and stability evaluation under thermal stress conditions at 4 °C and 40 °C.

The development of nanoliposomes was conducted according to protocols previously developed and standardized in our laboratory, ensuring methodological consistency and reproducibility. A schematic representation of the experimental workflow is provided in Figure 1, which illustrates the sequential stages of liposome preparation by ethanol injection, subsequent UHPH, and the LbL coating with chitosan of different molecular weights, followed by purification and physicochemical characterization. The detailed methodological description is presented in Section 2.3.1 and Section 2.3.2, corresponding respectively to the preparation of nanoliposomes and their surface modification with chitosan.

#### 2.3.1. Preparation of Nanoliposomes

Nanoliposomes were prepared using the ethanol injection method (Figure 1A), following procedures previously developed and standardized in our laboratory [25,31,32]. The organic phase was obtained by dissolving 3.0 g of soy lecithin in 50 mL of absolute ethanol to yield a 6% solution, while the aqueous phase consisted of phosphate-buffered saline (PBS) at pH 7.2 with ionic strength of 10 mM. The organic phase was injected into the aqueous phase at a rate of 1.5 mL/min using a peristaltic pump under continuous magnetic stirring at 300 rpm. The resulting dispersion was maintained under agitation for 10 min and subsequently diluted with PBS to a final volume of ~400 mL. A portion of the liposomal suspension was subjected to UHPH (Nano DeBEE, BEE International, South Easton, MA, USA) at 22,000 psi for four cycles, using six medium reactors and nozzle Z5 under reverse flow conditions. Homogenized (L-H) and non-homogenized (L-NH) liposomes were collected for subsequent stability assays. It is noteworthy that cholesterol was not incorporated into the preparation of these liposomal systems, despite its common use to provide rigidity and reduce permeability in vesicular bilayers [33,34]. In this study, the exclusion of cholesterol was intentional, as the primary objective was to evaluate the independent effect of UHPH on the lamellar structure formed solely by phospholipids. By omitting cholesterol, the structural contribution of the phospholipid matrix could be isolated, avoiding potential interference in the interpretation of homogenization-induced changes and ensuring a more accurate characterization of this technological variable in relation to liposomal stability and homogeneity.

#### 2.3.2. Layer-by-Layer Coating with Chitosan

Surface modification of nanoliposomes was performed using the LbL technique, also based on protocols previously established in our laboratory, Figure 1B [35,36]. Chitosan polymers of low, medium, and high molecular weight were weighed (1.7 mg each) and dissolved in 5 mL of 1% acetic acid under constant magnetic stirring. Subsequently, 5 mL of homogenized liposomes were added to each polymer solution and maintained under stirring at 300 rpm for 8 h to ensure adsorption of the polymer chains onto the liposomal surface. The coated systems were purified by ultrafiltration at 10,000 rpm for 6 min using polyethersulfone (PES) membranes with a pore size of 0.45 µm, thereby removing unbound polymer and ensuring reproducibility of the coating process [37].

#### 2.3.3. Physicochemical Characterization of Nanoliposomes

Particle size, polydispersity index (PDI), and zeta potential were determined for both homogenized and non-homogenized liposomes, before and after chitosan coating, using the same dynamic light scattering (DLS) and electrophoretic mobility protocols described previously in Section 2.2.1, Figure 1C. For liposomal dispersions, samples were diluted either in ultrapure water (≈5:5000, *v*/*v*) or PBS (1:16, *v*/*v*), depending on the assay, while zeta potential measurements were conducted in aqueous medium containing 0.035 mM NaCl to ensure ionic strength stability. All measurements were performed in triplicate, and results were expressed as mean ± standard deviation from freshly prepared dispersions to guarantee reproducibility and accuracy in the evaluation of colloidal stability.

### 2.4. Stability Study of Chitosan-Coated Nanoliposomes

The physical stability of liposomal systems was studied under constant storage conditions at 4 °C and 40 °C for a period of four weeks, Figure 1C. Particle size, polydispersity index (PDI), and zeta potential were measured at zero-time and subsequently at week four, where changes in colloidal stability were established. This approach allowed for a systematic evaluation of the influence of ultra-high-pressure homogenization (UHPH) and chitosan coating on the structural integrity of nanoliposomes under contrasting thermal environments.

### 2.5. Statistical Analysis

Data were tabulated and analyzed using the Minitab^®^ v. 17 software (Minitab^®^ Inc., State College, PA, USA). Statistical comparisons were made employing the ANOVA test, where the effects of UHPH treatment and molecular weight chitosan on the particle size, PDI, and potential zeta were evaluated. The Tukey post hoc test was used to determine the significance of differences between the independent groups and a confidence level of 95% was adopted.

## 3. Results and Discussion

### 3.1. Physicochemical Characterization of Chitosan in Aqueous Solution

**Figure 2:**
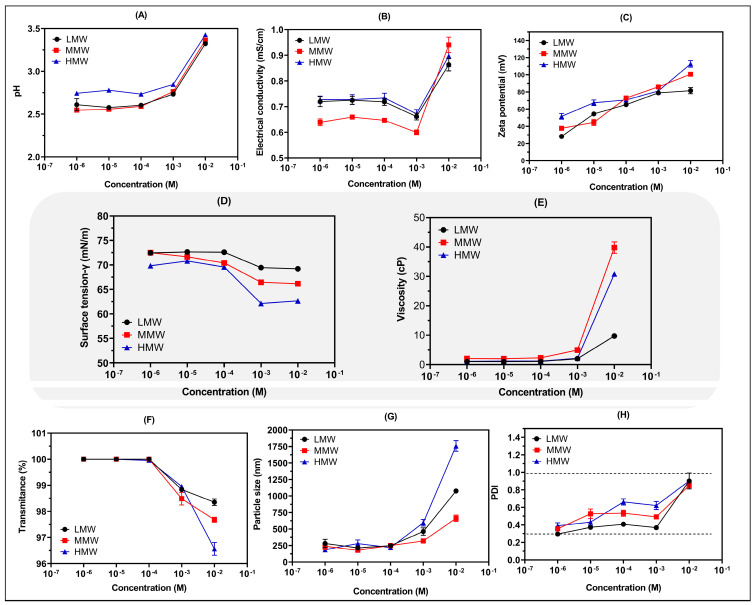
Physicochemical characterization of chitosan polymers with different molecular weights in aqueous medium in a concentration range of 10^−6^ to 10^−2^ M. (**A**) pH, (**B**) electrical conductivity, (**C**) zeta potential, (**D**) Surface tension, (**E**) viscosity, (**F**) Transmittance, (**G**) particle size, and (**H**) polydispersity index (PDI). LMW: low-molecular-weight chitosan; MMW: medium-molecular-weight chitosan; HMW: high-molecular-weight chitosan. Values are expressed as mean ± SD (n = 3).

The intrinsic physicochemical characterization of chitosan polymers with different molecular weights (LMW, MMW, HMW) in aqueous medium is presented in Figure 2.

The results presented in Figure 2A–C show the behavior of pH, electrical conductivity, and zeta potential as a function of polymer concentration (10^−6^–10^−2^ M). For pH and conductivity, the data display a concentration-dependent profile with a clear inflection point at 10^−3^ M, while zeta potential shows a linear increase. Regarding pH, it is observed that in the dilute regime between 10^−6^ and 10^−4^ M, values remain constant: around 2.5 for low- and medium-molecular-weight chitosan and 2.8 for high-molecular-weight chitosan. These results are consistent, since the pH is determined by the acidulated aqueous medium required to protonate the amino groups of chitosan, forming a polyelectrolyte system that allows solubilization in water [38]. Thus, when polymer concentration increases, the pH rises to about 3.5, indicating a decrease in hydronium ions. This decrease is attributed to the uptake of amino groups in a typical Henderson–Hasselbalch equilibrium for weak bases [39,40].

A similar result was observed for electrical conductivity, wherein the dilute regime values around 0.6–0.7 mS/cm were recorded, increasing to about 0.9 mS/cm, with an inflection point at 10^−3^ M. In contrast, zeta potential did not show this inflection but increased linearly in all three polymers. Linear regression analysis gave the following equations: Y = 5.695(x) + 114.12, R^2^ = 0.9161 for LMW; Y = 7.215(x) + 135.3, R^2^ = 0.9719 for MMW; and Y = 5.8977(x) + 131.03, R^2^ = 0.8933 for HMW. Although the R^2^ values are not close to 1 and do not indicate a perfect linear fit, a proportional trend between zeta potential and chitosan concentration can be established. All these results are consistent, since as molecular weight increases, the number of amino groups in the polymer chain also increases. This raises the charge fraction, producing a stronger electrical double layer and, consequently, a higher zeta potential [41].

Therefore, at low concentrations, both pH and electrical conductivity remain relatively stable, but at 10^−3^ M all polymers exhibit a notable increase, suggesting the formation of more complex ionic environments, possibly involving aggregated or reticulated configurations [42,43]. In contrast, zeta potential is directly linked to polymer concentration and molecular weight, as it reflects the effective charge density at the particle surface. Its nearly linear increase across the concentration range indicates the progressive accumulation of positive charges from protonated amino groups in the chitosan backbone. The steeper slope observed for MMW and HMW chitosan confirms that longer chains with higher charge density generate stronger electrostatic repulsion and enhanced colloidal stability. The divergence between conductivity and zeta potential is particularly relevant, since conductivity represents the contribution of free and mobile ions in the bulk solution, while zeta potential reflects the localized charge environment at the particle interface. The simultaneous increase in both parameters at 10^−3^ M marks the beginning of a cooperative regime, where chain proximity enhances charge redistribution and electrostatic interactions.

On the other hand, the results shown in Figure 2D,E describe the behavior of surface tension and viscosity as a function of polymer concentration. In both cases, a similar pattern is observed. In the dilute regime (10^−6^–10^−4^ M), both surface tension and viscosity remain practically constant, with values close to those of pure water. However, in the concentration range between 10^−4^ M and 10^−3^ M, a progressive decrease in surface tension and a slight increase in viscosity are observed, phenomena that intensify as the molecular weight of chitosan increases. Subsequently, in the most concentrated regime (10^−3^–10^−2^ M), surface tension remains constant, indicating that the air–water interface is saturated. From this point onward, the chitosan chains migrate toward the bulk, favoring the formation of cross-linked structures that significantly increase viscosity. Taken together, these results confirm that interface saturation occurs between 10^−4^ M and 10^−3^ M, and beyond this threshold, the system evolves towards configurations within the solution volume, a widely recognized behavior in polymeric systems [44,45].

Finally, the results shown in Figure 2F–H describe the optical and colloidal behavior of chitosan solutions through transmittance, particle size, and polydispersity index (PDI). In the dilute regime (10^−6^–10^−4^ M), all samples remain highly transparent, with transmittance values close to 100%, indicating a polymeric solution without significant light scattering. However, as chitosan concentration increases—particularly between 10^−4^ M and 10^−3^ M—transmittance begins to decrease, revealing the beginning of aggregation processes among polymer chains typical of concentrated regimes [42]. Furthermore, these changes are more pronounced for high-molecular-weight chitosan, which is consistent with the fact that larger particle sizes lead to stronger light-scattering effects [46]. In parallel, particle size increases progressively, confirming the formation of larger colloidal domains. Beyond 10^−3^ M, this growth becomes significant, suggesting cooperative association among polymer chains within the aqueous medium. Similarly, PDI values increase with chitosan concentration, evolving from narrow distributions typical of homogeneous dispersions to broader ones that reflect heterogeneous aggregation. This trend agrees with the transition from molecularly dispersed systems to crosslinked and viscous configurations. Taken together, these optical and light-scattering results demonstrate that around 10^−3^ M, chitosan undergoes a critical transition from a transparent, molecularly dispersed state to an aggregated regime. The magnitude of these changes is proportional to molecular weight, with high-molecular-weight chitosan exhibiting the greatest scattering, largest particle size, and highest PDI. These findings reinforce the interpretation derived from physicochemical and interfacial data, confirming that molecular weight governs the balance between electrostatic stabilization and structural association in chitosan solutions.

### 3.2. Physicochemical Characterization of Nanoliposomes Coated with Chitosan of Different Molecular Weights

Before discussing this section, it is important to note that cholesterol was not included in the development of these liposomal systems, since the objective was to evaluate the effect of UHPH on vesicular systems composed exclusively of phospholipids. The addition of cholesterol increases membrane rigidity, thereby interfering with this aim [47]. Nevertheless, under the experimental conditions employed, previous studies conducted in our laboratory had already demonstrated the formation of stable liposomes [25,36].

**Figure 3:**
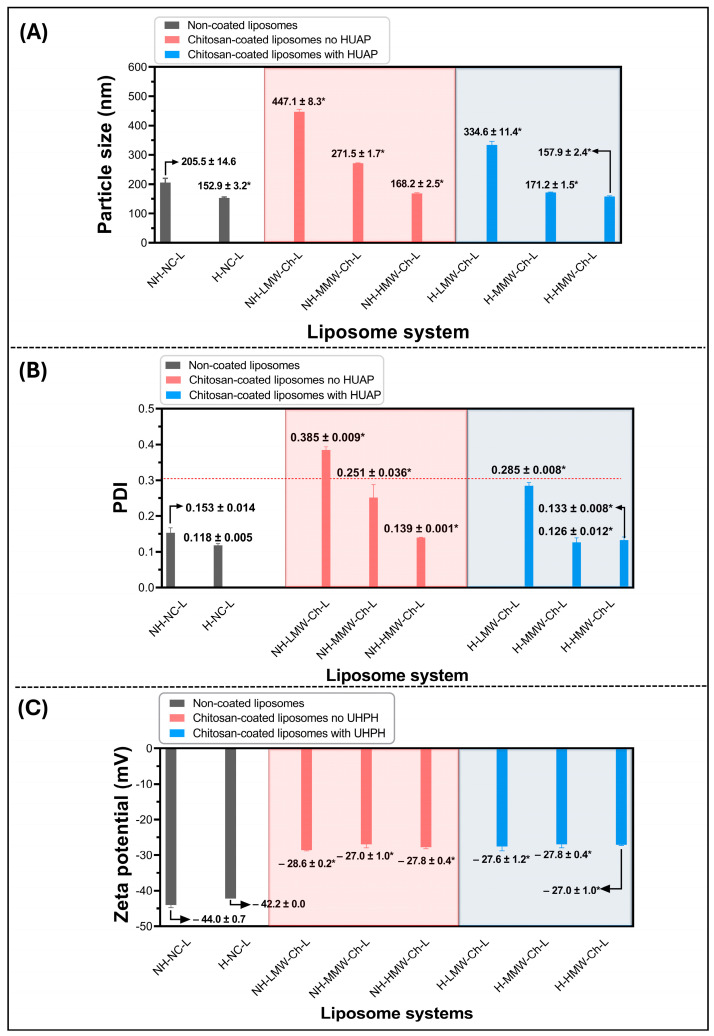
Physicochemical characterization of nanoliposomes. (**A**) Particle size, (**B**) polydispersity index (PDI), and (**C**) zeta potential of non-homogenized (NH) and homogenized (H) liposomes, either non-coated (NC) or coated with chitosan of low (LMW), medium (MMW), or high molecular weight (HMW). Values are expressed as mean ± SD (n = 3). Statistically significant differences are indicated (* *p* ≤ 0.005).

Figure 3 presents the particle size, polydispersity index (PDI), and zeta potential values of uncoated liposomes with and without UHPH treatment, as well as those coated with chitosan of different molecular weights. In the case of liposomes not homogenized by UHPH and without coating (NH-NC-L), the particle size was close to 205 nm, which decreased to approximately 153 nm (H-NC-L) after UHPH treatment (Figure 3A). This confirms the effectiveness of UHPH in producing smaller and more uniform vesicular structures [48,49,50,51]. The ~50 nm reduction can be attributed to the intense shear forces, cavitation, and turbulence generated during homogenization, a process that transitions from the rapid disorganization of multilamellar aggregates to a more ordered phospholipid self-assembly, ultimately yielding compact vesicles [51,52]. This size reduction is highly relevant for the development of nanosystems, as particle diameters below 200 nm have been reported to enhance colloidal stability and improve their performance as reservoirs of active compounds [53,54].

Regarding the PDI (Figure 3B), a similar trend was observed. Non-homogenized and uncoated liposomes (NH-NC-L) exhibited values of approximately 0.153, which decreased to ~0.118 (H-NC-L) after UHPH treatment, suggesting that particle size distribution tends to remain homogeneous. As for the zeta potential (Figure 3C), non-homogenized and uncoated liposomes (NH-NC-L) show negative values of ~−44 mV, which remained almost unchanged after UHPH treatment, reaching ~−42 mV (H-NC-L). These negative zeta potential values are ascribed to the phosphate groups of the phospholipids constituting the vesicular bilayer, a result widely documented in similar systems [48,55,56,57].

In the case of liposomal systems coated with chitosan, with or without UHPH treatment, the results revealed significant changes in particle size, polydispersity index (PDI), and zeta potential compared to the values described for uncoated liposomes. Regarding particle size, both non-homogenized and homogenized liposomes exhibited an increase, which was more pronounced in the non-homogenized systems. Interestingly, liposomes coated with low-molecular-weight chitosan displayed the largest particle sizes, reaching ~447 nm without UHPH (NH-LMW-Ch-L) and ~335 nm with UHPH (H-LMW-Ch-L). These were followed by the medium-molecular-weight chitosan systems, with values of ~272 nm without UHPH (NH-MMW-Ch-L) and ~172 nm with UHPH (H-MMW-Ch-L). In contrast, liposomes coated with high-molecular-weight chitosan exhibited the smallest particle sizes, ~168 nm without UHPH (NH-HMW-Ch-L) and ~158 nm with UHPH (H-HMW-Ch-L). A similar trend was observed for the PDI (Figure 3C). Systems coated with low-molecular-weight chitosan showed the highest values, ~0.385 without UHPH (NH-LMW-Ch) and ~0.285 with UHPH (H-LMW-Ch-L). Medium-molecular-weight chitosan systems followed, with ~0.251 without UHPH (NH-MMW-Ch-L) and ~0.126 with UHPH (H-MMW-Ch). Finally, liposomes coated with high-molecular-weight chitosan exhibited the lowest polydispersity, ~0.139 without UHPH (NH-HMW-Ch-L) and ~0.133 with UHPH (H-HMW-Ch-L). These findings are noteworthy, as they demonstrate that ultra-high-pressure homogenization exerts a clear effect on particle size and size distribution in liposomal systems. This observation is consistent with previous studies reporting that high-energy processes, such as UHPH or ultrasonic treatment, significantly influence particle size and distribution in both vesicular and nanoparticulate systems [58,59,60]. Indeed, it has been shown that if such processes are not properly optimized, they may lead to unfavorable outcomes, including destabilization or even degradation of these systems [61,62,63,64].

Regarding the zeta potential results (Figure 3C), polymer coating led to a moderate decrease in values, shifting from ~−42 mV to nearly constant values in the range of ~−27 mV to –29 mV. This observation is noteworthy, since appropriate coating is generally considered to occur when complete neutralization or charge inversion of the liposomal surface is achieved [65,66,67,68]. In this context, the moderate reduction suggests that chitosan adsorption onto the vesicular surface was neither complete nor uniform. When these findings are analyzed together with the changes in particle size and PDI, they reinforce the notion that, at the concentration tested, chitosan does not fully cover the liposomal surface but rather adsorbs onto specific regions. In the case of systems coated with low-molecular-weight chitosan, adsorption appears localized to certain surface areas, where multiple chains aggregate, leading to increased particle size and polydispersity, as shown in Figure 1B. In contrast, systems coated with medium- and high-molecular-weight chitosan may undergo a more compact and organized adsorption process without achieving complete coverage of the vesicular surface. It should be emphasized, however, that these representations are merely illustrative hypotheses. To elucidate the adsorption architecture in detail, additional studies focused on morphology and particle size are required, such as transmission electron microscopy (TEM), cryogenic TEM (cryo-TEM), atomic force microscopy (AFM), and nanoparticle tracking analysis (NTA), among others.

Finally, liposomal systems were also prepared following the sequence: (i) vesicle formation by ethanol injection, (ii) layer-by-layer chitosan coating, and (iii) subsequent UHPH treatment. Under these conditions, however, reproducible results could not be obtained, as DLS measurements consistently yielded low-quality signals characterized by large particle sizes and high polydispersity, translating into coarse colloidal populations and poor reproducibility across replicates. These findings point to a complete destabilization of the system and highlight the importance of exploring additional process variables, since UHPH performance is strongly influenced by factors such as fluid rate, applied pressure, nozzle size, number of cycles, and other operational parameters. Cho et al. reported that applying 21,800 psi and four UHPH cycles reduced the crystallinity and molecular weight of chitosan from 208 to 30 kDa [69]; Popa-Nita et al. described two ultrasound-induced depolymerization mechanisms, one leading to rapid chain scission and decreased polydispersity, and the other generating short chains and oligomers with increased polydispersity [70]; and Vargas et al. found a ~90% reduction in the consistency index of chitosan solutions after UHPH at 24,000 psi, accompanied by a transition from pseudoplastic to Newtonian flow behavior [71]. Therefore, these findings, reported by other researchers, could help explain the results obtained with chitosan-coated liposomes subsequently subjected to UHPH, highlighting the need for future studies focused exclusively on process optimization to establish the optimal conditions necessary to achieve robust and reproducible nanosystems under this sequence of coated liposome development.

### 3.3. Stability Study of Chitosan-Coated Nanoliposomes

**Figure 4:**
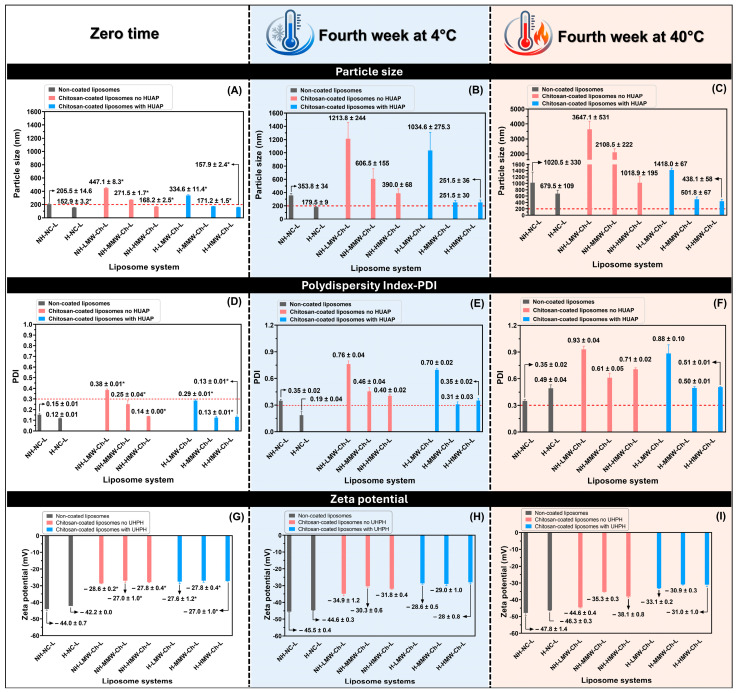
Stability study of nanoliposomal systems stored at 4 °C and 40 °C for four weeks. Panels (**A**–**C**) show changes in particle size, (**D**–**F**) in polydispersity index (PDI), and (**G**–**I**) in zeta potential for non-homogenized (NH) and ultra-high-pressure-homogenized (UHPH, H) liposomes, either non-coated (NC) or coated with chitosan of low (LMW), medium (MMW), or high molecular weight (HMW). Values are expressed as mean ± SD (n = 3). Statistically significant differences are indicated (* *p* ≤ 0.005).

In Figure 4, the results of changes in particle size, polydispersity index (PDI), and zeta potential are presented at zero time and after four weeks of storage under thermal stress conditions at 4 °C and 40 °C. However, since the initial physicochemical properties were already discussed in Section 3.2, the present analysis is mainly focused on the changes observed after four weeks of storage, highlighting the differences induced by temperature and polymer molecular weight of the coating.

In uncoated liposomal systems, a significant increase in particle size was observed. The initial values of ~205 nm for NH-NC-L and ~153 nm for H-NC-L (Figure 4A) increased to ~354 nm and ~179 nm, respectively, after four weeks of storage at 4 °C (Figure 4B); while under hot storage at 40 °C, they reached ~1021 nm and ~680 nm, respectively (Figure 4C). Similarly, the polydispersity index (PDI) also increased, rising from initial values of ~0.15 for NH-NC-L and ~0.12 for H-NC-L (Figure 4D) to ~0.35 and ~0.19 under cold storage (Figure 4E), and to ~0.35 and ~0.49 under hot storage (Figure 4F), respectively. In contrast, the zeta potential did not show considerable variations, as the initial values of ~−44 mV for NH-NC-L and ~−42 mV for H-NC-L (Figure 4G) changed only slightly to ~−46 mV and ~−45 mV under cold storage (Figure 4H), and to ~−48 mV and ~−46 mV under hot storage (Figure 4I), respectively. These results are consistent when considering that the liposomal formulations employed lacked stabilizing additives, making them intrinsically more vulnerable to aggregation. In this context, it is evident that UHPH contributes significantly to the stabilization of vesicular systems, maintaining particle sizes below 200 nm and PDI values < 0.3 under cold storage. However, under hot storage, destabilization intensifies, with size increases of nearly fivefold and a transition from slightly polydisperse systems to clearly multidisperse populations. This behavior, together with the slight increase in zeta potential, suggests the formation of multivesicular aggregates induced by the Ostwald aging process, where the rise in temperature and differences in vesicle size promote aggregation [72], in agreement with what was observed at lower temperatures, where this effect appears less pronounced.

Regarding liposomal systems coated with chitosan, marked differences were observed depending on UHPH treatment, polymer molecular weight, and storage temperature after four weeks. For non-homogenized systems stored at 4 °C, the change in particle size was significant. The NH-LMW-Ch-L system showed an increase of nearly threefold, reaching ~1214 nm, while NH-MMW-Ch L and NH-HMW-Ch-L doubled their initial sizes, reaching ~606 nm and ~390 nm, respectively (Figure 4B). Under hot storage at 40 °C, this effect was even more pronounced, maintaining the same trend with respect to chitosan molecular weight. In the case of NH-LMW-Ch L and NH-MMW-Ch-L, increases of nearly eightfold were observed, with values of ~3647 nm and ~2018 nm, respectively, while NH-HMW-Ch-L showed an increase of nearly sixfold, reaching ~1019 nm (Figure 4C). On the other hand, homogenized systems described a similar trend, although with slight improvement in stability. At 4 °C, the H-LMW-Ch-L system reached a particle size of ~1035 nm, equivalent to a threefold increase compared to the initial size, while H-MMW-Ch-L and H-HMW-Ch-L showed only slight changes, with increases of ~81 nm and ~94 nm, respectively, which did not correspond to even double the initial size (Figure 4B). For systems stored at 40 °C, H-LMW-Ch-L reached ~1418 nm, equivalent to a nearly fourfold increase over the initial size, while H-MMW-Ch-L and H-HMW-Ch L presented values of ~502 nm and ~438 nm, corresponding to size increases of approximately threefold (Figure 4C). These results suggest that as the molecular weight of chitosan increases, liposomal systems tend to stabilize more effectively, and this effect is enhanced when they have been previously subjected to UHPH. With respect to storage temperature, the findings are consistent in showing that destabilization intensifies at 40 °C, which may be attributed to desorption processes of chitosan from the vesicular surface, favoring the formation of multivesicular aggregates, polymeric chains, or both, ultimately increasing size populations [73]. Therefore, it could be indicated that vesicle development followed by UHPH treatment and subsequent coating with medium- and high-molecular-weight chitosan constitutes an interesting strategy to preserve vesicular integrity under thermal stress.

These results are reinforced by the analysis of the polydispersity index (PDI), which showed behavioral patterns like those described for particle size. In systems coated with low-molecular-weight chitosan, the initial PDI was ~0.38 in non-homogenized (NH-LMW-Ch-L) and ~0.29 in homogenized (H-LMW-Ch-L) systems (Figure 4D). After four weeks of storage, these values increased significantly, reaching ~0.76 and ~0.70 under cold storage, and ~0.93 and ~0.88 under hot storage, respectively (Figure 4E,F). In contrast, vesicular systems coated with medium- and high-molecular-weight chitosan showed lower polydispersity values, as well as more moderate increases under thermal storage. In non-homogenized systems, NH-MMW-Ch-L and NH-HMW-Ch-L increased from ~0.25 and ~0.14 (Figure 4D) to ~0.46 and ~0.40 under cold storage, and to ~0.61 and ~0.71 under hot storage, respectively (Figure 4E,F). Meanwhile, homogenized systems, H-MMW-Ch-L and H-HMW-Ch-L, maintained even more stable values, with slight increases and narrower distributions, rising from ~0.13 (Figure 4D) to ~0.31 and ~0.35 under cold storage, and to ~0.51 and ~0.51 under hot storage (Figure 4E,F).

In contrast to particle size and PDI, zeta potential did not show considerable variations, as mentioned at the beginning of this section. At zero time, uncoated liposomal systems presented values of ~−44 mV and ~−42 mV for NH-NC-L and H-NC-L, respectively, while coated systems with and without UHPH oscillated between ~−27 and ~−29 mV (Figure 4G). After four weeks of storage, the changes remained slight in relation to thermal stress. Under cold storage, uncoated systems maintained values close to ~−45 mV, regardless of homogenization, while coated systems showed ranges of ~−30 to ~−35 mV in non-homogenized and ~−28 to ~−29 mV in homogenized systems (Figure 4H). Under hot storage, uncoated systems remained between ~−46 and ~−49 mV, while coated systems reached values of ~−38 to ~−45 mV in non-homogenized and ~−31 to ~−33 mV in homogenized systems (Figure 4I). These results indicate that, despite the increase in particle size and the emergence of populations with different dimensions, the systems continue to behave like colloids capable of establishing an electrical double layer. This character is maintained mainly due to the formation of vesicular structures, which preserve the negative surface potential attributed to the phosphate groups present in phospholipids, one of the major components of the raw material used for the preparation of vesicular systems.

## 4. Conclusions

This study demonstrated that the chitosan polymers employed, characterized by a high degree of deacetylation (75–85%) and three different molecular weights—low (50–190 kDa), medium (190–310 kDa), and high (310–375 kDa)—were solubilized in acidulated aqueous solutions. A transition concentration between 1 × 10^−4^ and 1 × 10^−3^ M was identified, where chitosan shifted from a state of free-chain dissolution to a regime of multichain attraction.

Regarding liposome preparation, the sequence consisting of (i) ethanol injection, followed by (ii) ultra-high-pressure homogenization (UHPH) and subsequently (iii) layer-by-layer coating with chitosan polymers proved more convenient than the alternative sequence of ethanol injection, coating, and subsequent UHPH. The latter route led to complete destabilization of the nanocolloidal system, suggesting that if this pathway is to be pursued, new configurations or optimization of UHPH conditions would be required.

When liposomal systems were subjected to UHPH prior to coating, vesicles exhibited smaller sizes (~150 nm), narrower size distributions (~0.1), and highly negative zeta potential values (~−42 mV), features attributable to improved colloidal stability against aggregation. With respect to coating, the polymer amounts were insufficient to achieve complete surface coverage. Low-molecular-weight chitosan produced systems with larger particle sizes and higher polydispersity, whereas medium- and high-molecular-weight chitosan generated comparatively more compact and less polydisperse systems. These effects were accompanied by a decrease in zeta potential, plausibly due to depolarization arising from layer-by-layer interactions between chitosan and the liposomal surface.

Storage at 40 °C intensified destabilization, promoting chitosan desorption and Ostwald diffusion processes that triggered aggregation. This effect was most pronounced in systems coated with low-molecular-weight chitosan, followed by medium, and finally high-molecular-weight chitosan, which provided the most effective coating. Under cold storage, even in the absence of stabilizing additives, high-molecular-weight chitosan maintained vesicular systems at around ~250 nm with polydispersity values of ~0.3–0.4 and zeta potentials of ~−20 mV.

These findings support the hypothesis that polymer molecular weight and homogenization sequence critically influence vesicular stability. However, further studies are required to provide direct morphological evidence of coating architecture, for example through microscopy, and to clarify the mechanisms of chitosan adsorption. It is also necessary to evaluate different chitosan concentrations beyond 10^−3^ M to determine whether zeta potential inversion can be achieved and sustained. Finally, optimization studies of UHPH conditions are warranted, particularly for the sequence involving ethanol injection, coating, and subsequent homogenization, and future work should test more robust formulations incorporating stabilizing components such as cholesterol, surfactants, or additional polymers.

## Data Availability

The original contributions presented in this study are included in the article. Further inquiries can be directed to the corresponding authors.

## Abbreviations

The following abbreviations are used in this manuscript:

LbL	Layer-by-layer
UHPH	Ultra-high-pressure homogenization
L-H	Homogenized liposomes
NL-H	Non-homogenized liposomes
LMW	Low-molecular-weight chitosan
MMW	Medium-molecular-weight chitosan
HMW	High-molecular-weight chitosan
NH-NC-L	Non-homogenized, non-coated liposomes
H-LMW-Ch-L	Homogenized low-molecular-weight chitosan-coated liposomes
H-MMW-Ch-L	Homogenized medium-molecular-weight chitosan-coated liposomes
H-HMW-Ch-L	Homogenized high-molecular-weight chitosan-coated liposomes
NH-LMW-Ch-L	Non-homogenized low-molecular-weight chitosan-coated liposomes
NH-MMW-Ch-L	Non-homogenized medium-molecular-weight chitosan-coated liposomes
NH-HMW-Ch-L	Non-homogenized high-molecular-weight chitosan-coated liposomes
